# Supplementary material for: Rad51-mediated replication of damaged templates relies on monoSUMOylated DDK kinase
Source: Nat Commun. 2022 May 5;13:2480. doi: 10.1038/s41467-022-30215-9 (PMC9072374; doi:10.1038/s41467-022-30215-9)
Supplement: Supplementary file 1 — Supplementary Information [file 41467_2022_30215_MOESM1_ESM.pdf]

Rad51-mediated replication of damaged templates relies on monoSUMOylated DDK kinase

Chinnu Rose Joseph[1], Sabrina Dusi[1], Michele Giannattasio[1,2] and Dana Branzei[1,3*]

[1]IFOM, Istituto Fondazione di Oncologia Molecolare, Via Adamello 16, 20139 Milan, Italy

[2]Università degli Studi di Milano, Dipartimento di Oncologia ed Emato-Oncologia, Via S. Sofia 9/1, 20122 Milano, Italy

[3]Istituto di Genetica Molecolare, Consiglio Nazionale delle Ricerche (IGM-CNR), 27100 Pavia, Italy

[*] Corresponding author

E-mail: dana.branzei@ifom.eu

The authors declare no conflicts of interest

Supplementary Figures and legends

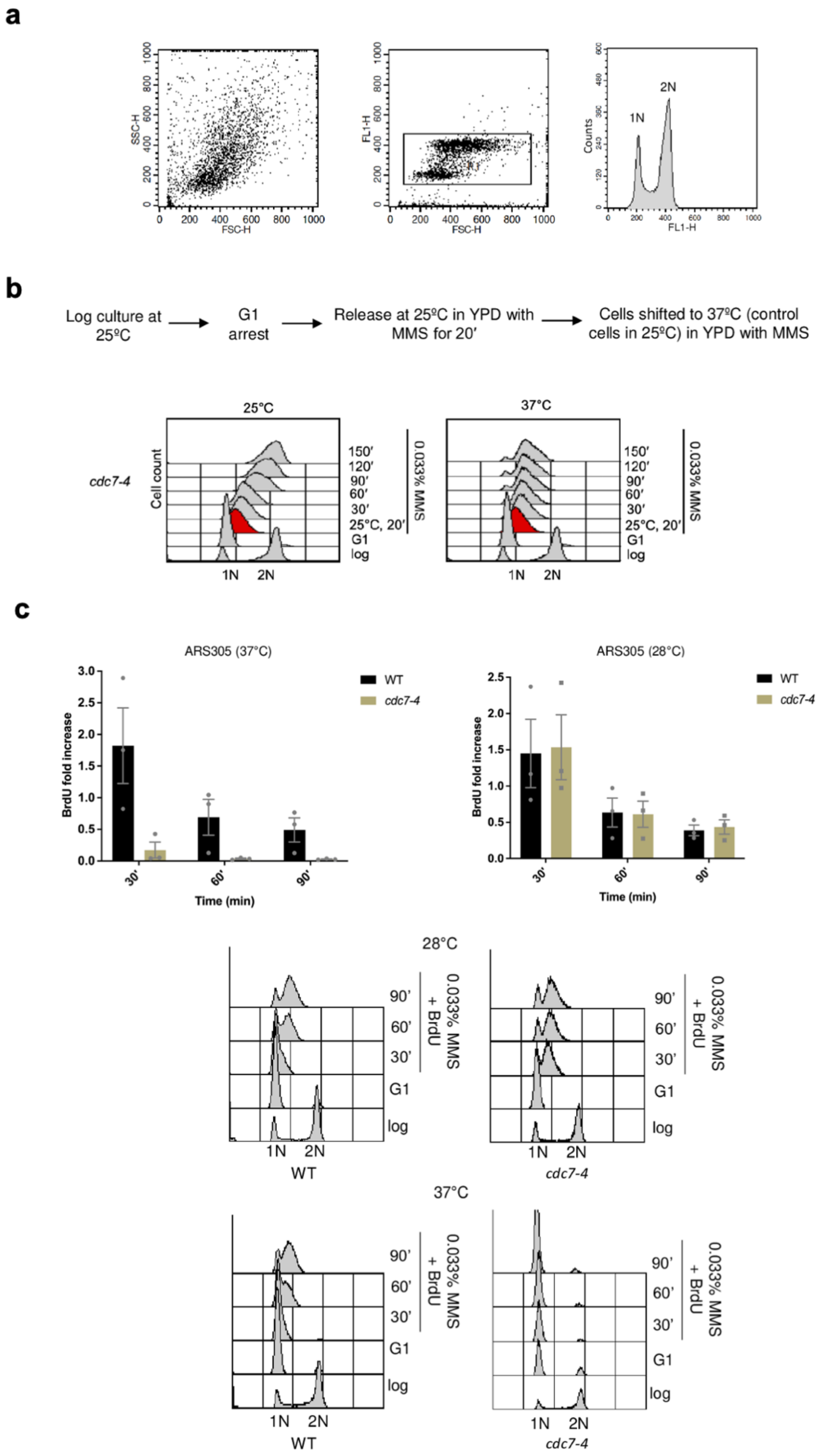

Supplementary Fig. 1. Experimental Conditions that Allow Temperature Sensitive *cdc7-4* Mutants to Progress in S Phase. (a) Gating strategy of Flow cytometry and a representative image of FACS. To exclude all the debris and doublets, samples were gated on FSC-H and SSC-H as well as on FSC-H and FL1-H. Then a histogram of FL1-H was generated from the remaining cells. A value of 200 in FL1-H represents G1 population of cells with 1N DNA content and a value of 400 represents G2 population of cells with 2N DNA content. 50,000 cells were analyzed per each sample for each experiment. (b) Exponential cells were synchronized in G1 phase using alpha factor and released at permissive temperature (25°C) for the initial 20 min, then the cells were either shifted to non-permissive temperature (37°C) to inactivate Cdc7-4 or kept at 25°C all throughout the experiment. Samples for FACS were collected every 30 min. 1N and 2N below the graph indicate the DNA content of cells in G1 and G2/M phases of the cell cycle, respectively. (c) (top) BrdU-IP qPCR profiles for wildtype (WT) and *cdc7-4* cells released from G1 arrest into S phase in the presence of 0.033% MMS and 200 μg/ml BrdU for indicated timepoints at 37°C and 28°C. BrdU incorporation was analyzed at the ARS305 locus and the fold increase was calculated as a ratio: IP/Input. Each ChIP experiment was performed three times and each real time PCR was performed in triplicates. Error bars represent the standard error of mean SEM. (below) FACS analysis to monitor the cell cycle progression for BrdU-qPCR indicated above. Exponential cells were synchronized in G1 phase using alpha factor and released in a semi-permissive temperature 28°C or kept at 37°C. Samples for FACS were collected every 30 min. 1N and 2N below the graph indicate cells in G1 and G2/M phases of the cell cycle, respectively.

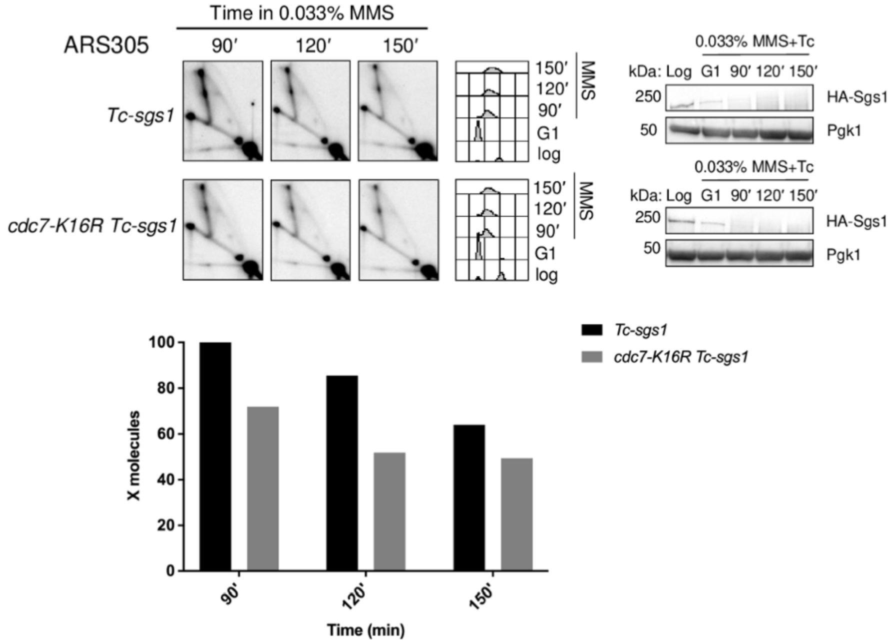

Supplementary Fig. 2. MonoSUMOylated Cdc7 promotes replication-associated recombination. 2D gel analysis of recombination intermediates extracted from cells of the indicated genotype in two independent experiments. Experimental set up is as described in Figure 1a. 1 mM tetracycline was added during G1 arrest and then cells were released in the presence of 1 mM tetracycline. Levels of HA-tagged Sgs1 were detected by western blotting analysis with Pgk1 serving as loading control.

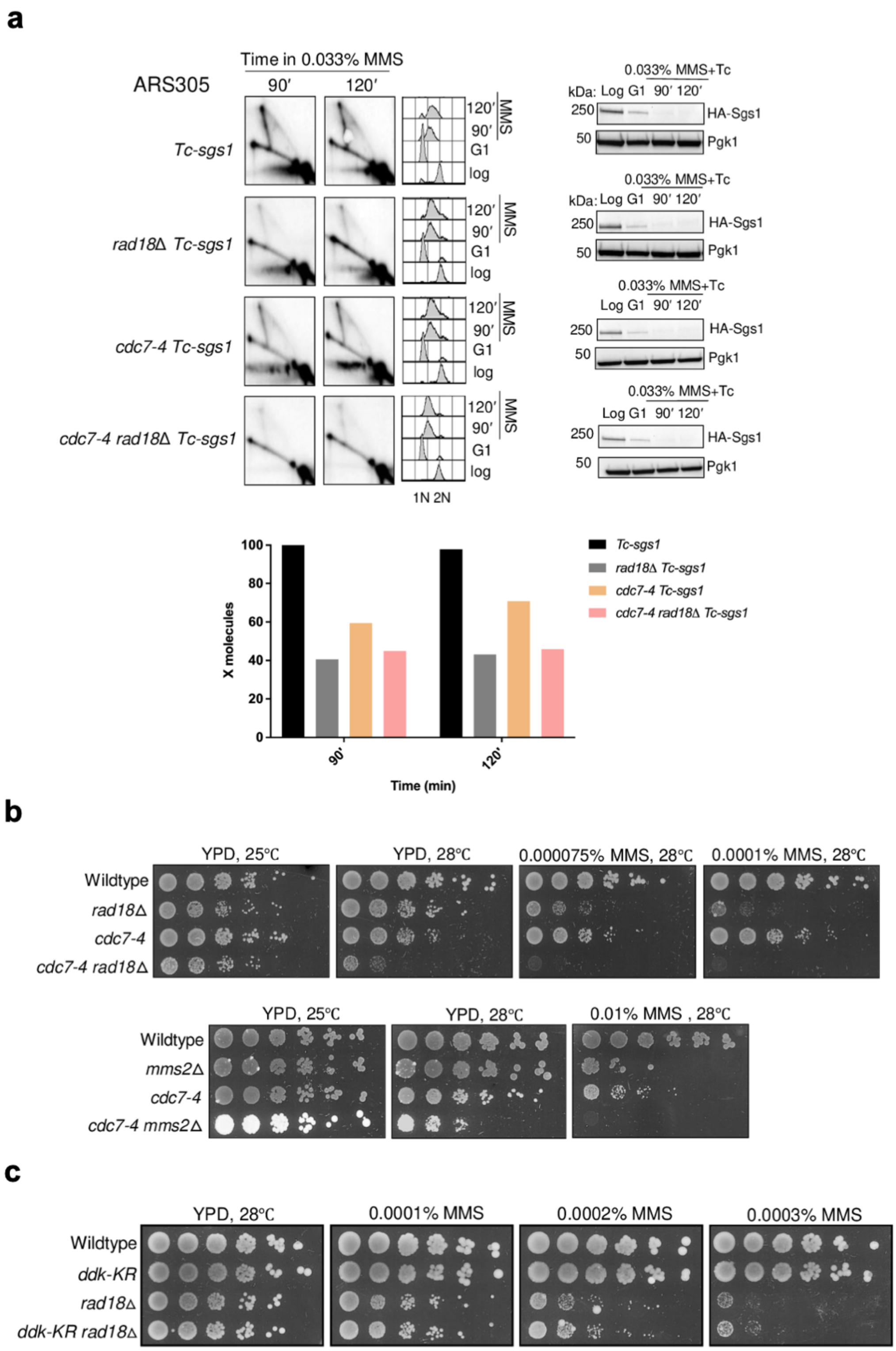

Supplementary Fig. 3. DDK acts jointly with PCNA polyubiquitylation in gap-filling.

(a) Exponentially growing *Tc-sgs1*, *rad18*Δ *Tc-sgs1*, *cdc7-4 Tc-sgs1* and *cdc7-4 rad18*Δ *Tc-sgs1* cells were arrested in G1 phase at 25°C and released into medium containing 0.033% MMS and tetracycline (1 mM) at 28°C. Samples were collected at the indicated time points for

2D gel, western blotting and FACS analysis in two independent experiments. Samples for 2D were *in vivo* crosslinked with psoralen followed by genomic DNA extraction and digestion with *Nco*I. The 2D gel signals were analyzed for ARS305. Depletion of HA-Sgs1 was visualized by western blotting with Pgk1 serving as loading control. Cell cycle progression of each strain was monitored by analyzing the FACS plots. The relative enrichment of X-shaped replication intermediates is represented in the histograms. In the graph, signal intensities were normalized with respect to the monomer spot and the highest value obtained during quantification of the X molecule was assigned as 100%. (b, c) Exponentially grown cultures of the indicated strains were adjusted to the same concentration, serially diluted ten-fold and spotted on YPD plates for the indicated MMS concentrations. Plates were incubated at 28°C and photographed after 2 days.

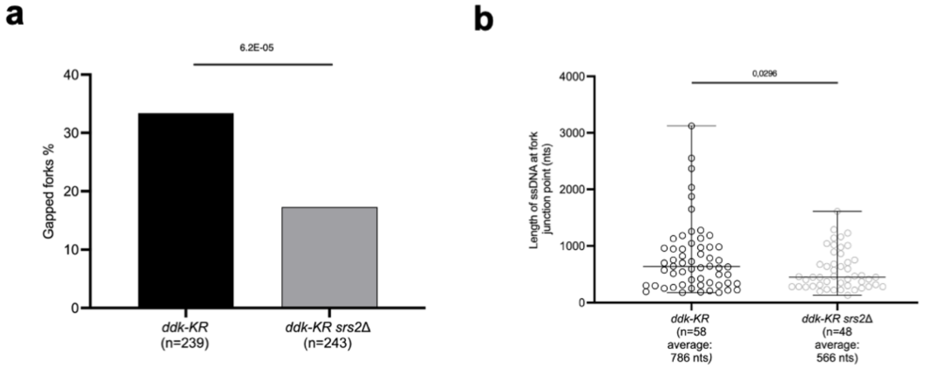

Supplementary Fig. 4. Activation of salvage recombination through loss of Srs2 suppresses fork uncoupling. (a) TEM analysis of the DNA replication intermediates in the indicated strains exposed to MMS. Experimental setup is the same as described in Fig. 5c. Histogram represents the percentage of gapped replication forks generated in *ddk-KR srs2*Δ mutants in comparison with *ddk-KR*. Total number (n) of DNA replication-recombination intermediates analyzed for each genotype is indicated. The indicated P-values were calculated by Fischer's exact two-sided test. (b) Scatter dot plot represents the distribution of length of the ssDNA gaps (in nucleotides) at the fork branching points of *ddk-KR srs2*Δ mutants in comparison with *ddk-KR*. Middle line in the scatter dot plot indicates the average value of the length of the ssDNA discontinuity, bars represent distribution range. P-value was calculated by unpaired two-tailed Student's t-test.

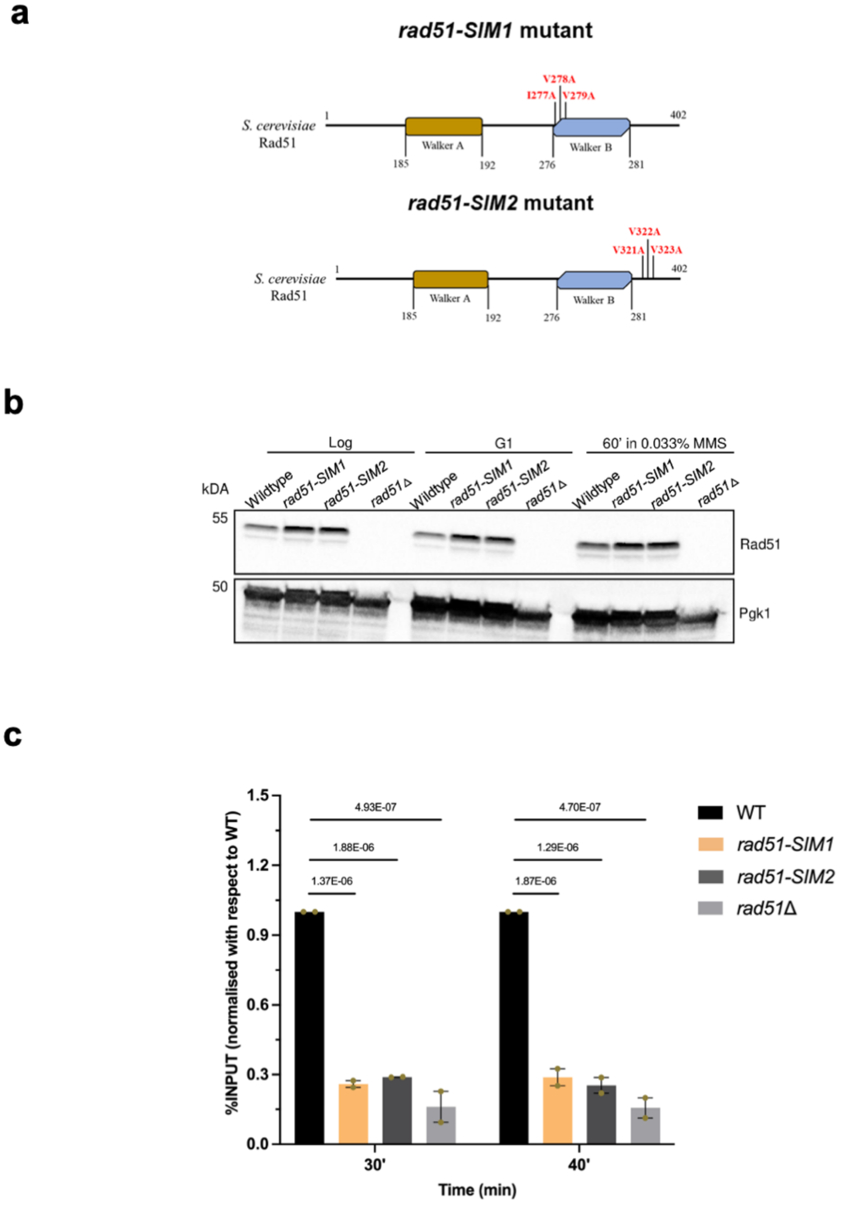

Supplementary Fig. 5. Illustration of Rad51 putative SIMs and engineered mutations. (a) Schematic representations of Rad51 depicting the putative SIMs and the engineered mutations. *rad51-SIM1* (Top), *rad51-SIM2* (Bottom). (b) Western blotting analysis to study Rad51 protein stability in wildtype, *rad51-SIM (rad51-SIM1 and rad51-SIM2)* and *rad51Δ* cells. Exponentially grown cells were arrested in G1 phase and then released in YPD medium containing 0.033% MMS for 60 min. Samples were collected at the indicated timepoints and Rad51 was detected by western blot in two independent experiments. Pgk1 served as the

loading control. (c) Exponentially grown cultures of the indicated strains were adjusted to the same concentration, serially diluted (1:10) and then spotted on YPD plates with the indicated drug concentrations. Plates were scanned after 2 days of spotting. (d) Rad51 binding at early ARS305 replication origin was analyzed n the indicated yeast strains, using chromatin immunoprecipitation coupled to quantitative PCR (ChIP-qPCR). Exponentially growing WT, *rad51-SIM1*, *rad51-SIM2* and *rad51*Δ cells were arrested in G1 phase at 25°C and released into 0.033% MMS-containing YPD medium at 28°C. Samples for the ChIP-qPCR analysis were collected at 30 min and 40 min from the G1 release into S-phase in the presence of MMS. The experiment was repeated two times and each real time PCR was performed in triplicates. The histogram reports the enrichment signals expressed as percentage of the signal in the corresponding input sample. The enrichment values obtained were then normalized and expressed as fractions of the enrichment obtained in the wild type cells, which was considered as 1. Error bars represent the SEM (mean value +/- standard error of mean) of two independent experiments. The indicated p-values were calculated by 2-way ANOVA test.

Supplementary Table 1: *Saccharomyces cerevisiae* Strains Used in This Study.

Strain	Genotype	Source
FY1296	Mata *ade2-1 trp1-1 leu2-3,112 his3-11,15 ura3 can1-100 Rad5*+ (W303)	Lab collection
HY4017	W303 Mata *Tc-sgs1::KanMX*	Lab collection
HY6842	W303 Mata sgs*1Δ::HIS3*	Lab collection
FY1143	W303 Mata *cdc7-4*	Lab collection
HY7881	W303 Mata *cdc7-4*	This study
HY7599	W303 Mata *cdc7-4 Tc-sgs1::KanMX*	This study
HY10899	W303 Mata *sgs1Δ::NAT cdc7-as3*	This study
FY1110	W303 Mata *ura3::URA3/GPD-TK(7X)*	Lab collection
HY 9284	W303 Mata *cdc7-4 ura3::URA3/GPD-TK(7X)*	This study
HY0923	W303 Mata *mms2Δ::HPHMX6*	Lab collection
HY8775	W303 Mata *mms2Δ:: HPHMX6 cdc7-4*	This study
HY0139	W303 Mata *rad18Δ::HPHMX4*	Lab collection
HY8296	W303 Mata *rad18Δ::LEU2 cdc7-4*	This study
HY8207	W303 Mata *rad18Δ::LEU2 sgs1::pADH1-tc3-3xHA-Sgs1 (KanMX)*	This study
HY8209	W303 Mata *rad18Δ::LEU2 cdc7-4 sgs1::pADH1-tc3-3xHA-Sgs1 (KanMX)*	This study
HY8298	W303 Mata *mms2Δ::HPHMX6 sgs1::pADH1-tc3-3xHA-Sgs1 (KanMX)*	This study
HY8379	W303 Mata *cdc7-4, sgs1::pADH1-tc3-3xHA-Sgs1 (KanMX) mms2Δ::HPHMX6*	This study

HY6645	W303 Mata *siz1Δ::HPH*	Lab collection
HY8778	W303 Mata *siz1Δ::HPH cdc7-4*	This study
HY8351	W303 Mata *siz1Δ::HPH sgs1::pADH1-tc3-3xHA-Sgs1 (KanMX)*	This study
HY8354	W303 Mata *siz1Δ::HPH cdc7-4 sgs1::pADH1-tc3-3xHA-Sgs1 (KanMX)*	This study
HY11386	W303 Mata *ddk-KR siz1Δ::HPH*	This study
HY10785	W303 Mata *ddk-KR siz1Δ Tc-sgs1*	This study
FY1987	W303 Mata *cdc7as3MYC bar1Δ::HisG*	Lab collection
HY10899	W303 Mata *cdc7as3MYC bar1Δ::HisG sgs1Δ::NAT*	This study
HY9123	W303 Mata *pCDC7-cdc7-K16R pDBF4-dbf4-K6R,K14R,K432R*	Lab collection
HY10323	W303 *(LEU2)pCDC7-cdc7-K16R (HIS3)pDBF4-dbf4-K6R,K14R,K432R(hphNT1) sgs1::pADH1-tc3-3xHA-Sgs1 (KanMX)*	This study
HY3845	W303 Mata *ARS305* sgs1::pADH1-tc3-3xHA-Sgs1(KanMX).*	Lab collection
HY10608	W303 Mata *ARS305* sgs1::pADH1-tc3-3xHA-Sgs1(KanMX) cdc7-4*	This study
HY10718	W303 Mata *ARS305* sgs1::pADH1-tc3-3xHA-Sgs1(KanMX) (LEU2)pCDC7-cdc7-K16R(HIS3)pDBF4-dbf4-6R,K14R,K432R(hphNT1)*	This study
HY10624	W303 Mata (LEU2) pCDC7-cdc7-K16R *sgs1::pADH1-tc3-3xHA-Sgs1 (KanMX)*	This study

HY11287	W303 Mata *cdc7-4 mre11-H125N*	This study
HY9590	W303 Mata *cdc7-4 exo1Δ::HIS3*	This study
HY7295	W303 Mata rad51Δ::URA	Lab collection
HY11701	W303 Mata *pCDC7-cdc7-K16R pDBF4-dbf4-K6R,K14R,K432R rad51Δ::LEU2*	This study
HY10813	W303 Mata *pCDC7-cdc7-K16R pDBF4-dbf4-K6R,K14R,K432R mms2Δ::NAT*	This study
HY12359	W303 Mata pCDC7-cdc7-K16R pDBF4-dbf4-K6R,K14R,K432R rad18Δ::LEU2	This study
HY12066	W303 Mata *Rad51:: rad51-I277A/V278A/V279A -HPX*	This study
HY12068	W303 Mata *Rad51:: rad51-V321A/V322A/V323A -HPX*	This study
HY0992	W303 Mata rad51Δ::LEU2 sgs1Δ::NATMX4	Lab collection
HY12107	W303 Mata *rad51:: rad51-I277A/V278A/V279A -HPX sgs1Δ*::HIS3	This study
HY12111	W303 Mata *rad51:: rad51-V321A/V322A/V323A -HPX sgs1Δ*::HIS3	This study

Supplementary Table 2. Reagents and oligos used in this study.

Reagent	Source	Identifier
**Antibodies**		
Anti-HA mouse monoclonal (12CA5) (Dilution for western blot 1:2000)	ThermoFisher	Cat#MA1-12429
Anti-Pgk1 mouse monoclonal (22C5D8) (Dilution for western blot 1:5000)	Invitrogen	Cat# **459250**
Anti-mouse IgG, HRP-linked antibody (Dilution for western blot 1:20000)	Biorad	Cat#1706516
Anti-BrdU	MBL	MI-11-3
Anti-Rad51 (Dilution for western blot 1:2000)	Santa Cruz Biotechnology	sc 33626 (y-180)
Anti-Rad51	ThermoFisher	Cat# PA5-31144
**Chemicals, peptides, and recombinant proteins**		
alpha-factor mating pheromone (WHWLQLKPGQPMY)	GenScript; RRID: SCR_002891	Cat# 59401-28-4
Nocodazole	Sigma-Aldrich	Cat# M1404
Methyl Methane Sulfonate	TCI-Europe	Cat#66-27-3
Ribonuclease A from bovine pancreas	Sigma-Aldrich	Cat# R5503
SYTOX™ Green Nucleic Acid Stain	ThermoFisher	Cat#S7020
Proteinase K, recombinant, PCR Grade	Roche	Cat# 03115801001
Formaldehyde solution	Sigma-Aldrich	Cat# 47608
Complete, EDTA-free protease inhibitor cocktail tablets	Roche	Cat# 4693132001
Quantifast SYBR green PCR kit	Qiagen	Cat# 1300000559
Dynabeads Protein G	Invitrogen	Cat# 1003D
Zymolyase 100T (*Arthrobacter luteus*)	Seikagaku Corporation	Cat# 120493
4,5',8-Trimethylpsoralen	Sigma-Aldrich	Cat# T6137
GeneScreen Hybridization Transfer Membrane	Perkin-Elmer	Cat#NEF983001PK
Agarose D1-LE	Fisher Molecular Biology	Cat# AS-101
Prime-a-Gene labeling system	Promega	Cat# U1100
EasyTide dCTP [α-32P] 6000Ci/mmol 20mCi/ml	Perkin-Elmer	N/A
ProbeQuant G-50 micro columns	GE Healthcare	Cat# 28903408
NcoI-HF	New England BioLabs	Cat#R3193L
EcoRV-HF	New England BioLabs	Cat#R3195L
Bromodeoxyuridine	Sigma-Aldrich	Cat# B2883-100MG
Tetracycline	Nzy tech	Cat# MB02202
L-Canavanine	Sigma-Aldrich	Cat#C1625
Benzoylated Naphthoylated DEAE-Cellulose	Sigma-Aldrich	Cat#B6385
Poly-Prep chromatography column	Biorad	Cat#7311550
Amicon Ultra-0.5 ml 100K	Merck Millipore	Cat#UFC510096
**Critical commercial assays**		
QIAquick PCR purification kit	QIAGEN	Cat# 28106
Criterion TGX stain-free Precast Gels	Biorad	Cat#5678093
Deposited data		
Raw and analyzed data	This paper	Excel file

Experimental models: Organisms/strains		
All yeast Saccharomyces cerevisiae strains used in this work are W303 background derivatives with the wild type RAD5+ locus. They are listed in Table S1.	This paper (see Table S1)	N/A
**Oligonucleotides**		
5′-TCAGAGCCTTCTTTGGAGCT-3′	This paper (qPCR)	ARS305_3FW
5′-TCACACCGGACAGTACATGA-3′	This paper (qPCR)	ARS305_3RV
5' GTTCCGAAACAGGACACTTAGC-3'	This paper (2D)	305Fw
5' ATCCAGGAGGGACTCAATGTAG-3'	This paper (2D)	305Rv
5′-GAGCGAGTCTCGGTTTTCCTTGGC TGCGGCCGATTCTGTTATGGCTCT ATAC-3′	This paper (For creation of Rad51-SIM1)	Rad51 SIM1 FP1
5′-GTATAGAGCCATAACAGAATCGG CCGCAGCCAAGGAAAACCGAGACT CGCTC-3′	This paper (For creation of Rad51-SIM1)	Rad51 SIM1 RP1
5′-ACCAATTTGGTGTTGCAGCCGCCG CTACTAACCAAGTGGTCGC-3′	This paper (For creation of Rad51-SIM2)	Rad51 SIM2 FP1
5′-GCGACCACTTGGTTAGTAGCGGCG GCTGCAACACCAAATTGGT-3′	This paper (For creation of Rad51-SIM2)	Rad51 SIM2 RP1
