# Supplementary material for: Rad51-mediated replication of damaged templates relies on monoSUMOylated DDK kinase
Source: Nat Commun. 2022 May 5;13:2480. doi: 10.1038/s41467-022-30215-9 (PMC9072374; doi:10.1038/s41467-022-30215-9)
Supplement: Supplementary file 3 — Reporting summary [file 41467_2022_30215_MOESM3_ESM.pdf]

nature portfolio

Corresponding author(s): Dana Branzei

Last updated by author(s): March 21, 2022

Reporting Summary

Nature Portfolio wishes to improve the reproducibility of the work that we publish. This form provides structure for consistency and transparency in reporting. For further information on Nature Portfolio policies, see our Editorial Policies and the Editorial Policy Checklist.

Statistics

For all statistical analyses, confirm that the following items are present in the figure legend, table legend, main text, or Methods section.

n/a	Confirmed	
☐	☒	The exact sample size (*n*) for each experimental group/condition, given as a discrete number and unit of measurement
☐	☒	A statement on whether measurements were taken from distinct samples or whether the same sample was measured repeatedly
☐	☒	The statistical test(s) used AND whether they are one- or two-sided<br>*Only common tests should be described solely by name; describe more complex techniques in the Methods section.*
☒	☐	A description of all covariates tested
☐	☒	A description of any assumptions or corrections, such as tests of normality and adjustment for multiple comparisons
☐	☒	A full description of the statistical parameters including central tendency (e.g. means) or other basic estimates (e.g. regression coefficient) AND variation (e.g. standard deviation) or associated estimates of uncertainty (e.g. confidence intervals)
☐	☒	For null hypothesis testing, the test statistic (e.g. *F*, *t*, *r*) with confidence intervals, effect sizes, degrees of freedom and *P* value noted<br>*Give P values as exact values whenever suitable.*
☒	☐	For Bayesian analysis, information on the choice of priors and Markov chain Monte Carlo settings
☒	☐	For hierarchical and complex designs, identification of the appropriate level for tests and full reporting of outcomes
☒	☐	Estimates of effect sizes (e.g. Cohen's *d*, Pearson's *r*), indicating how they were calculated

Our web collection on statistics for biologists contains articles on many of the points above.

Software and code

Policy information about availability of computer code

Data collection	BIORAD Image Lab Version 5.2.1 for Western Blot acquisition<br>Amersham Typhoon Scanner software V1.0 for 2D gel acquisition<br>BD CellQuest Version 3.3 for FACS sample collection<br>Roche LightCycler 96 version 1.1.0.1320 for qPCR
Data analysis	BIORAD Image Lab Version 5.2.1 for quantification of Western Blots<br>ImageJ 1.50i for preparation of 2D gel pictures<br>BD CellQuest Version 3.3 for FACS analysis<br>Numbers version 10.3.5 (7029.5.5) for ChIP-qPCR analysis

For manuscripts utilizing custom algorithms or software that are central to the research but not yet described in published literature, software must be made available to editors and reviewers. We strongly encourage code deposition in a community repository (e.g. GitHub). See the Nature Portfolio guidelines for submitting code & software for further information.

Data

Policy information about availability of data

All manuscripts must include a data availability statement. This statement should provide the following information, where applicable:

- Accession codes, unique identifiers, or web links for publicly available datasets
- A description of any restrictions on data availability
- For clinical datasets or third party data, please ensure that the statement adheres to our policy

The authors declare that all data supporting the findings of this study are available within the paper and its supplementary information files. The source data

underlying all figures, Figures 1-7 and Supplementary Figs. 1-5, are provided in the Mendeley datasets (DOI: 10.17632/c284kmxv6x.1).Joseph, Chinnu Rose; Dusi, Sabrina ; Giannattasio, Michele; Branzei, Dana (2022), “SUMOylated DDK Prevents Fork Uncoupling and Promotes Rad51-Mediated Replication of Damaged Templates ”, Mendeley Data, V1, doi: 10.17632/c284kmxv6x.1

Field-specific reporting

Please select the one below that is the best fit for your research. If you are not sure, read the appropriate sections before making your selection.

☒ Life sciences ☐ Behavioural & social sciences ☐ Ecological, evolutionary & environmental sciences

For a reference copy of the document with all sections, see nature.com/documents/nr-reporting-summary-flat.pdf

Life sciences study design

All studies must disclose on these points even when the disclosure is negative.

Sample size	No statistical methods were used to predetermine sample size, as this study did not include animal models or human participants. Sample size was determined based on the gold standards in the field and experiments to obtain statistical significance and reproducibility. We used 2000 million cells for each 2D gel point, 20 000 cells for each flow cytometry analysis point, 10 million cells for each ChIP sample, over 100 molecules for EM analysis).
Data exclusions	No data were excluded
Replication	All experimental findings were reliably reproduced as indicated in the figure legends. All experiments were repeated at least 2 times to ensure reproducibility. We have not experienced cases of non-reproducible data in this study. At least two biological replicates were included for ChIP-qPCR. The mean values of 3(or 2) experiments were calculated and shown with corresponding SEM. Statistical analyses were performed using a two-way ANOVA testt. 2D gel experiments were repeated at least 2 times and in some cases additionally different alleles, timepoints or regions were used to confirm the results.
Randomization	No randomization was done because this study did not involve animals or human participants. Samples were organized into groups based on treatment and genotype. Appropriate controls were included in all experiments.
Blinding	Before each experiment, the yeast strains that were used were given numbers instead of the genotype or condition and the numbers were then connected to the strains only after analysis

Reporting for specific materials, systems and methods

We require information from authors about some types of materials, experimental systems and methods used in many studies. Here, indicate whether each material, system or method listed is relevant to your study. If you are not sure if a list item applies to your research, read the appropriate section before selecting a response.

Materials & experimental systems

n/a	Involved in the study	
☐	☒	Antibodies
☐	☒	Eukaryotic cell lines
☒	☐	Palaeontology and archaeology
☒	☐	Animals and other organisms
☒	☐	Human research participants
☒	☐	Clinical data
☒	☐	Dual use research of concern

Methods

n/a	Involved in the study	
☒	☐	ChIP-seq
☐	☒	Flow cytometry
☒	☐	MRI-based neuroimaging

Antibodies

Antibodies used	anti-BrdU (MBL, cat. no: MI-11-3)<br>anti-Rad51 (rabit polyclonal, Santa Cruz Biotechnology, sc 33626 (y-180)) used for ChIP, Western blot 1:2000<br>anti-HA (mouse monoclonal, clone 12CA5, Invitrogen cat. no: MA1-12429) used in Western Blot 1:2000<br>anti-Pgk1 (mouse monoclonal, clone 22C5D8, Invitrogen; cat. no: 459250) used in Western Blot 1:5000
Validation	All antibodies in this study were used for Western Blot analysis in S. cerevisiae yeast samples and the bands for the respective proteins corresponded with the expected size. The application (Western Blot) and species (S. cerevisiae) were indicated on the manufacturers websites.<br>We have validated anti-HA aantibody in Western Blot by using an S. cerevisiae yeast strain without any tag to confirm specificity. The Pgk1 antibody is commonly used as a loading control in many publications (Gay et. al., 2018).

Eukaryotic cell lines

Policy information about cell lines

Cell line source(s)	Yeast W303
Authentication	The strains were confirmed with resistant markers, PCR, sequencing, western blotting wherever relevant.
Mycoplasma contamination	NA
Commonly misidentified lines (See ICLAC register)	NA

Flow Cytometry

Plots

Confirm that:

☒ The axis labels state the marker and fluorochrome used (e.g. CD4-FITC).

☒ The axis scales are clearly visible. Include numbers along axes only for bottom left plot of group (a 'group' is an analysis of identical markers).

☒ All plots are contour plots with outliers or pseudocolor plots.

☒ A numerical value for number of cells or percentage (with statistics) is provided.

Methodology

Sample preparation	For flow cytometry analysis, approximately 1.5x107 cells for each timepoint were collected and fixed in 70% ethanol. Cells were suspended in 10 mM Tris pH 7.5 buffer, and RNA and proteins were removed by RNaseA (0.4 mg/ml) and proteinase K (1 mg/ml) treatment. Subsequently, cells were stained in SYTOX green solution (1μM) and analyzed using a FACSCalibur Flow Cytometer.
Instrument	BD FACSCalibur Flow Cytometer
Software	BD CellQuest Version 3.3
Cell population abundance	No populations were sorted, only cell cycle progression was followed by analyzing 20.000 cells per sample
Gating strategy	Samples were gated on SSC-H and FSC-H as well as on FL1-H and FSC-H to exclude doublets and debris. Then a histogram of FL1-H values was generated from the remaining cells. A value of 200 in FL1-H represents the G1 population with a 1N DNA content and a value of 400 represents the G2 population with a 2N DNA content.

☒ Tick this box to confirm that a figure exemplifying the gating strategy is provided in the Supplementary Information.
